# How Do Movement Patterns in Weightlifting (Clean) Change When Using Lighter or Heavier Barbell Loads?—A Comparison of Two Principal Component Analysis-Based Approaches to Studying Technique

**DOI:** 10.3389/fpsyg.2020.606070

**Published:** 2021-01-25

**Authors:** Inge Werner, Nicolai Szelenczy, Felix Wachholz, Peter Federolf

**Affiliations:** ^1^Department of Sport Science, University of Innsbruck, Innsbruck, Austria; ^2^BFF Training Ulm, Ulm, Germany

**Keywords:** weightlifting, clean, principal component analysis PCA, technique analysis in sport, motion patterns, principal movements

## Abstract

This study compared whole body kinematics of the clean movement when lifting three different loads, implementing two data analysis approaches based on principal component analysis (PCA). Nine weightlifters were equipped with 39 markers and their motion captured with 8 Vicon cameras at 100 Hz. Lifts of 60, 85, and 95% of the one repetition maximum were analyzed. The first PCA (PCA^trial^) analyzed variance among time-normed waveforms compiled from subjects and trials; the second PCA (PCA^posture^) analyzed postural positions compiled over time, subjects and trials. Load effects were identified through repeated measures ANOVAs with Bonferroni-corrected *post-hocs* and through Cousineau-Morey confidence intervals. PCA^trial^ scores differed in the first (*p* < 0.016, η*_*p*_*^2^ = 0.694) and fifth (*p* < 0.006, η*_*p*_*^2^ = 0.768) principal component, suggesting that increased barbell load produced higher initial elevation, lower squat position, wider feet position after squatting, and less inclined arms. PCA^posture^ revealed significant timing differences in all components. We conclude, first, barbell load affects specific aspects of the movement pattern of the clean; second, the PCA^trial^ approach is better suited for detecting deviations from a mean motion trajectory and its results are easier to interpret; the PCA^posture^ approach reveals coordination patterns and facilitates comparisons of postural speeds and accelerations.

## Introduction

Weightlifting and corresponding movements are often used for muscle power training in jumping or sprinting (Bolger et al., 2016; Hackett et al., 2016; Berton et al., 2018). The power clean is part of muscle power training as well as modern forms of recreational muscle training and attracts scientific attention. Load recommendations for explosive force training and muscle adaptation are discussed in the literature (McBride et al., 2011; Fisher et al., 2017; Ammar et al., 2018), however, studies investigating adaptations in movement technique to differing loads are rare (Winchester et al., 2005; Hadi et al., 2012).

For learning and perfecting the correct lifting technique, movements are also performed using low loads. At a first glance, no differences in the movement patterns are expected when lifting lower loads, because sequencing of muscle activation, relative time of muscle activation, and relative force of lifting muscles are expected to be constant (Schmidt and Lee, 2019). In contrast, modern theories of motor control (Todorov and Jordan, 2002) point out that movement variability is controlled when and where the goal of the movement is at risk. Accordingly, if a maximum of force output is the desired task goal, variability of muscle activation is suggested to be dramatically reduced (Cohn et al., 2018). When lifting lower weights, one may expect less refined adaptations to achieve the task goal and therefore more inconsistent movement patterns. While training with lower loads has the advantage of allowing for more repetitions, it may on the other hand, carry the risk of practicing movement patterns that are not optimal for lifting close to-maximal or maximal loads.

In weightlifting two different techniques are distinguished: the snatch and the clean and jerk. Both techniques start with movement sequences called first pull, transition phase and second pull (Enoka, 1979; Garhammer, 1984), whereas the sequences turnover under the barbell, catch and stand up into end position differ between the two techniques. Studies investigating weightlifting technique often focus on barbell movement patterns or power output, but lack information on movements of all body segments (Gourgoulis et al., 2009; Kipp and Meinerz, 2017). It could be shown that increasing lifting loads leads to decreasing maximal barbell height in the clean and snatch techniques as well as lower maximal vertical velocity of the barbell (McBride et al., 2011; Hadi et al., 2012; Ammar et al., 2018). However, analyzing only few selected variables (e.g., peak position, velocity, and acceleration of the barbell or of individual body segments) might be insufficient to map complex movement strategies. The current study focuses on the clean-movement. By applying an analysis technique that considers the movements of all body segments, it revisits the question if and how increasing weight affects the movement patterns, i.e., the technique, during the clean.

To extract information from the waveforms of relevant variables or for determining movement synergies during the whole movement, the usage of principal component analysis (PCA) has gained popularity. The method reduces dimensionality of datasets by detecting correlations of waveforms and/or by decomposing complex whole-body movements into sets of one-dimensional movement components (Daffertshofer et al., 2004). This method was already applied in research on weightlifting to assess leg and pelvis movements or barbell acceleration patterns (Kipp et al., 2012; Kipp and Harris, 2015). These studies report that conducting the clean exercise with different loads revealed no effect of barbell load on kinematic patterns. However, these results are based on leg and pelvis positions but did not evaluate trunk and arm movements.

Sorting through movement studies applying PCA revealed two frequent approaches to construct the input data matrix: first, the input matrix may consist of trial vectors incorporating all variables (e.g., marker positions) and how they evolve over a normed time period (columns) collected over all subjects and trials (rows) (Brandon et al., 2013; Federolf et al., 2013a; Kobayashi et al., 2016; Nordin and Dufek, 2016; Zago et al., 2017a; Rossi et al., 2018). The PCA then reveals patterns of correlating deviations from the mean motion deriving from all subjects and trials (PCA^trial^). Second, the input matrix may consist of posture vectors, e.g., defined by the position of markers placed on all body segments (columns) collected over time, subjects, and trials (rows). The PCA then finds patterns of correlations within the variations of posture vectors as they change over time (PCA^posture^) and maps them on movement components/movement synergies (Maurer et al., 2012; Federolf et al., 2014; Hsiu-Hui et al., 2016; Majed et al., 2017; Gløersen et al., 2018; Promsri et al., 2018; Wachholz et al., 2020). To the best of our knowledge, no comparison between these two approaches in application of PCA for technique analysis in sports has been completed so far. Such a comparison is of interest to rank approaches according to their explanatory power in use for strength and conditioning coaches and scientists.

The aims of the current study were 2-fold. First, the study investigated the hypothesis that barbell load influences the movement pattern of the clean. This is consequential for training, since lower barbell load allows for more repetitions, but, if the hypothesis is correct, would entail the risk of acquiring movement patterns that might be sub-optimal for maximal loads.

A second purpose of the current study was the evaluation of advantages and disadvantages between the two data analysis approaches. Both approaches involve a PCA calculation to structure kinematic movement patterns, however, the first approach bases the PCA on time-normalized trial vectors; the second approach is based on posture vectors.

## Methods

### Participants

A convenient sample of 11 weightlifters of diverse skill levels and age volunteered for the study. The test protocol was approved by the Board for Ethical Questions in Science of the University of Innsbruck (Certificate 42/2015). All participants signed an informed consent form prior to the measurements.

Two datasets had to be discarded because sweating led to loss of markers or because the required number of trials were not completed due to a minor injury. Hence, data of nine weightlifters could be analyzed in the current study (age 27.2 ± 13.8 years; weight 69.3 ± 16.6 kg; height 166.1 ± 11.0 cm; one repetition maximum (1 RM) 122.2 ± 18.9% of body weight; mean ± standard deviation).

### Measurement Procedures

After an individual, self-selected warming up, 12 clean repetitions were performed: 5 with 60%, 4 with 85%, and 3 with 95% of the weightlifter’s competition one repetition maximum (1 RM), which was self-reported by the athletes. A trial was successful, if the movement met weightlifting rules and ended in upright position with the bar fixed on the shoulder. According to this definition two trials had to be repeated throughout the measurements. The rest period between the 60%-lifts was 90 s. After a rest of 5 min the participants performed the 4 cleans at 85% 1 RM with a break of 120 s between lifts. After another 5 min rest period, the participants completed the three trials at 95% 1 RM, again with 120 s breaks between each lift. Again, this sequence matches the procedures that these weightlifters were used to in their usual training sessions.

### Data Collection

The participants were equipped with 39 markers according to the plug-in-gait marker placement scheme (Davis et al., 1991): 4 head markers, cervical spine C7, thoracic spine T10, clavicular (positioned under the fossa jugularis sterni) and sternum (processus xiphoideus), right back (reference marker, right scapula); pairwise left and right: shoulder (acromion), upper arm, elbow (lateral epicondyle), wrist in- and outside, finger (knuckle of the index finger), pelvic front (spina iliaca anterior superior) and pelvic back (spina iliaca posterior superior), thigh, knee (epicondylus lateralis femoris), shank, ankle (malleolus lateralis), heel (tuber calcanei), and toe (basis of the bunion). Two additional markers were placed on the left and right end of the lifting bar. The bar was accordant to the norm of IWF (International Weightlifting Federation) and had a mass of 20 kg. During the clean movements, athletes were recorded with 8 VICON cameras (Vicon motion systems, Oxford, United Kingdom) at a sampling rate of 100 Hz.

#### Data Processing

Markers were labeled using Nexus 2.5 software (Vicon Motion Systems Ltd.). Due to the large changes in posture during squatting, the sweating of some participants, the explosive movement, and reflections present during some measurements, trials were affected by gaps in the marker trajectories. Some gaps could be filled using a custom gap filling algorithm (Federolf, 2013; Gløersen and Federolf, 2016), nevertheless sub-sets of trials were selected for further analysis: 3 trials of the 60% 1 RM cleans, and 2 trials of each, the 85% 1 RM and the 95% 1 RM cleans. Data were further analyzed using Matlab (R2012a, The MathWorks^TM^, Natick, MA, United States). From each trial, a sequence starting with the first displacement of the bar and ending 30 frames into the stand-up phase after the catch was extracted for further analysis. The marker on the right back (one sided reference marker) and both finger markers (often lost due to high accelerations) were discarded. The remaining 36 marker positions were centered and normalized with the mean Euclidean distance (e.g., Federolf et al., 2013b; Zago et al., 2017b; Haid et al., 2019; Verheul et al., 2019).

**FIGURE 1:**
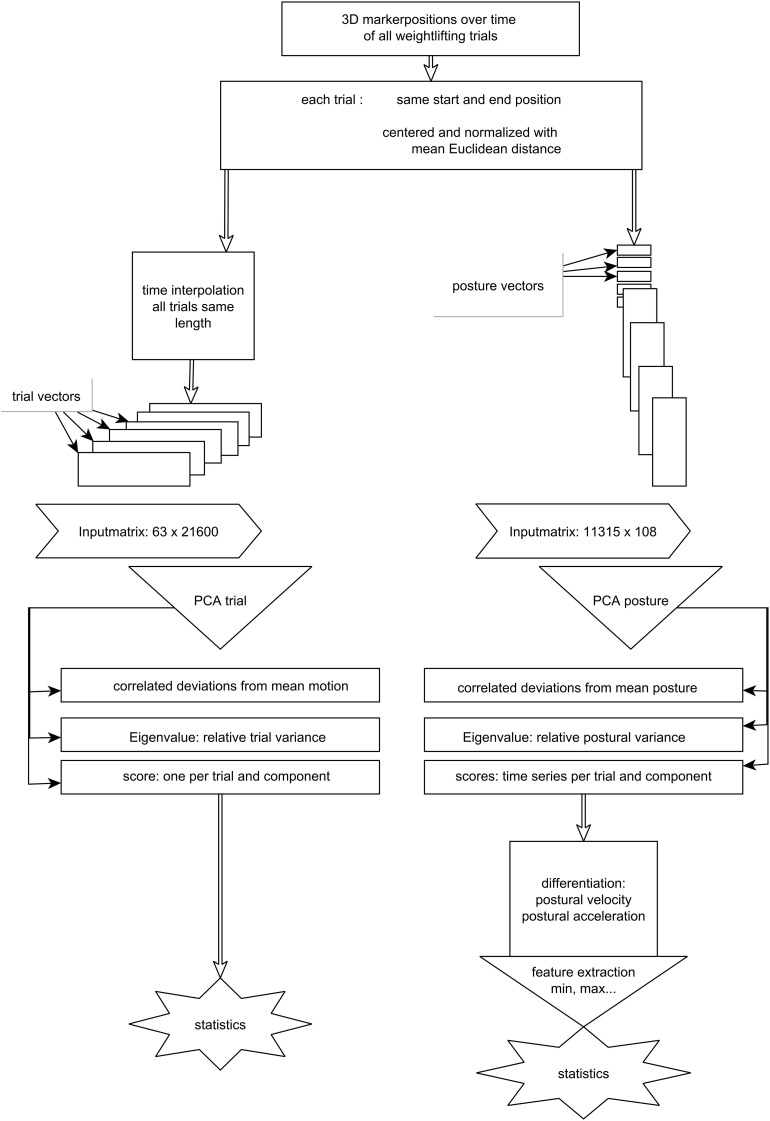
Overview of the data processing steps.

Two different approaches to conduct a PCA were implemented using custom written Matlab codes (Figure 1). For the first approach, PCA^trial^, all trials were time-normalized to 200 frames. These trial vectors for all markers (columns) were transformed into single rows for each subject and each trial (PCA input matrix: rows = 7 trials^∗^9 volunteers; columns = 36 markers^∗^3D^∗^200 frames; centered by subtracting mean of each column). PCA then results in one score per trial, per eigenvector (Federolf et al., 2013a). These scores quantify for each trial, how much the variable waveforms deviated from the mean waveforms over all trials according to the pattern defined by each eigenvector.

For the second approach, PCA^posture^, each trial was represented as a matrix of posture vectors, where a posture vector is all marker positions at a certain time point. These trial matrices were normalized and concatenated (Gløersen et al., 2018) to analyze the variance between posture vectors with the PCA^1^ (PCA input matrix: rows = duration of trial^∗^sampling frequency^∗^7 trials^∗^9 volunteers; columns = 36 markers^∗^3D; centered by subtracting subject mean posture and normalized to mean Euclidean distance as described in Federolf, 2016 or Gløersen et al., 2018). PCA^posture^ results in time series of scores called principal (postural) positions PP (Federolf, 2016; Haid et al., 2018). The score time series were differentiated to obtain postural velocity, PV, and postural acceleration, PA, components (Federolf, 2016; Haid et al., 2018, 2019; Promsri et al., 2018; Longo et al., 2019; Zago et al., 2019; Promsri and Federolf, 2020; Wachholz et al., 2020).

### Statistics

Eigenvalues of each PCA were expressed as percentage of explained variance. For both PCAs the first 8 principal components were considered. PCA^*trial*^ scores were averaged for the trials of the same person and the same condition (60, 85, and 95% lifts). These mean scores were tested for normality using the Shapiro-Wilk test. If normality was confirmed, repeated measures ANOVAs (Pillai trace) were conducted separately for all components to reveal score differences depending on load conditions. Direct comparisons of load conditions were conducted where the rANOVA showed significance using paired samples *t-*tests. If the Shaprio Wilk test manifested non-normality, Friedman and Wilcoxon tests were performed.

PCA^posture^ explained variance was calculated from the scores of each principal component. The PCA^posture^ scores are time series, i.e., waveforms representing the postural changes during the trial. Mean waveforms were calculated to represent each load condition of each volunteer. For the subject mean waveforms, Cousineau-Morey confidence intervals were calculated to identify non-overlapping phases (Cousineau, 2005; Baguley, 2012). Additionally, maxima and minima of the postural position (these are highest and lowest scores over time), postural velocity and postural acceleration were determined and differences between load conditions were analyzed using rANOVAs. Effect sizes are reported as partial eta squared and Cohen’s d for dependent samples. For reliability measurements, ICCs of scores over time in the same load condition were computed.

All statistical calculations were done in SPSS (IBM, version 24) and the level of significance was set to α < 0.05, Bonferroni corrected in *post hoc* analyses.

## Results

### PCA^trial^

**FIGURE 2:**
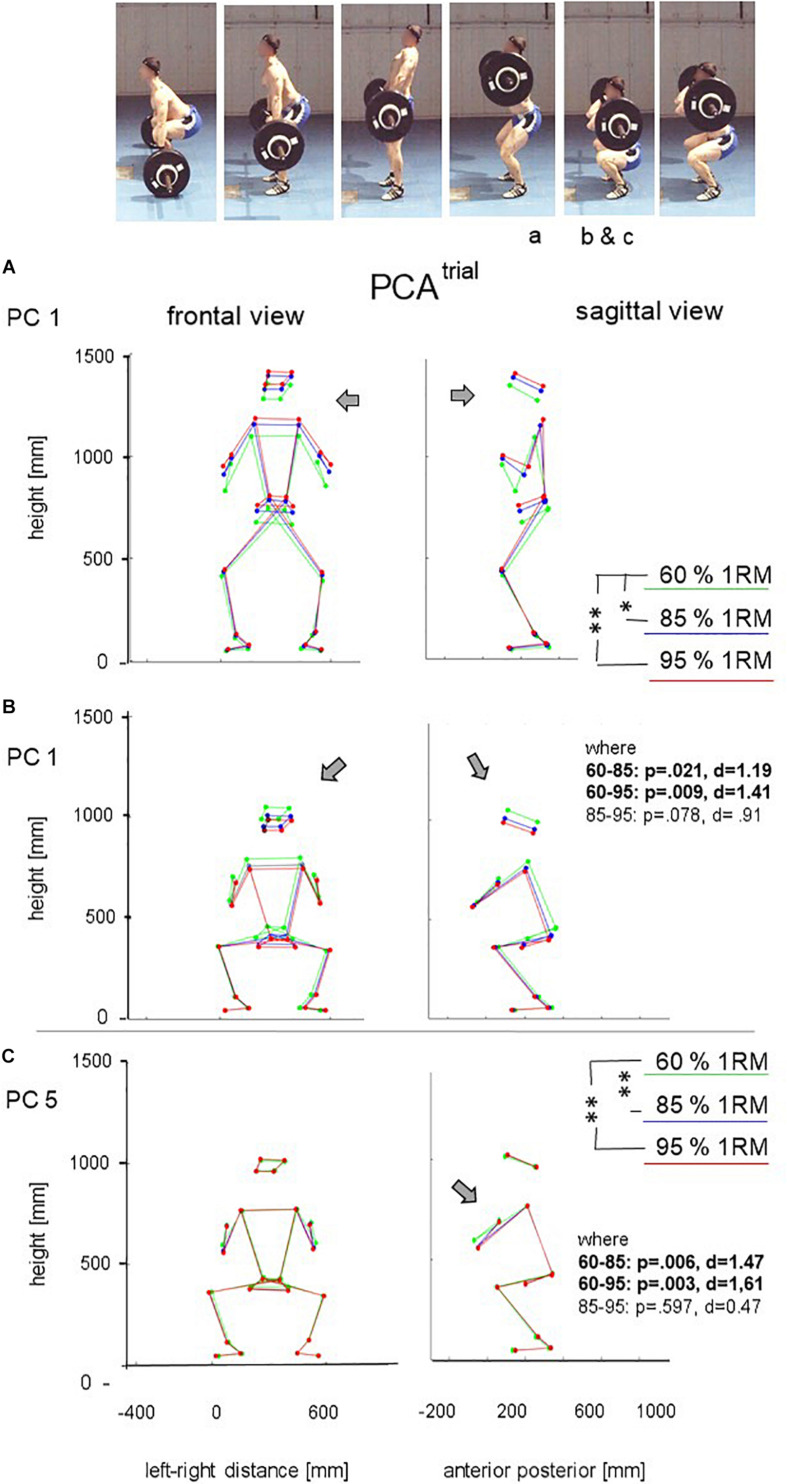
Sagittal and frontal view at highest and lowest bar position of PC1 and at lowest bar position of PC5 for 60, 85, and 95% of 1 RM load. **(A)** mean position of PC1 at the moment of maximal bar height; **(B)** mean position of PC1 at the moment of the minimal bar height in the catching phase; **(C)** mean position of PC5 at the moment of minimal bar height in the catching phase.

The first 8 PCs explained 85% of the variance between trials (Supplementary Table 1 and Supplementary Videos 1, 2) provide an overview of what aspects of variation were quantified by the PC components. Repeated measures ANOVA revealed significant effects of load on the scores of PC1 [*F*(2, 7) = 7.94, *p* = 0.016, η*_*p*_*^2^ = 0.694] and PC5 [*F*(2, 7) = 11.60, *p* = 0.006, η*_*p*_*^2^ = 0.768]. *Post hoc* tests showed differences between 60 and 85% or 95% of 1 RM but not between 85 and 95% lifts. Figure 2 shows selected frames (highest barbell position in the mean motion before squat; lowest barbell position during squat), for which the effects were visualized: increased barbell load led to a higher body position in stance (Figure 2A) and a lower body position with wider foot placement in the squat (Figure 2B). PC5 captured knee and hip extension in the onset of the second pull, the position of the elbows during squat (Figure 2C) and indicated higher and wider elbow positions particularly in 60% 1 RM compared to the higher loads.

### PCA^posture^

**FIGURE 3:**
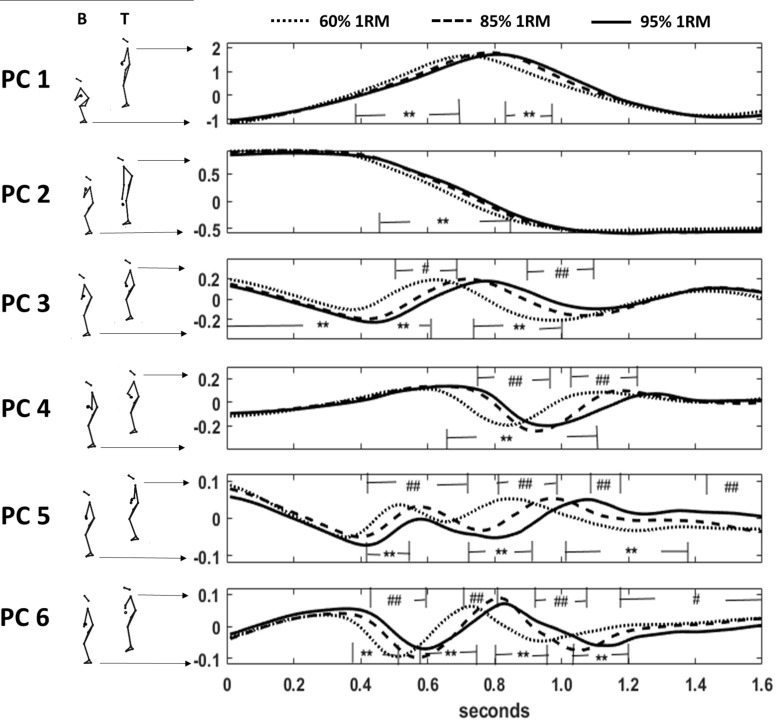
Scores over time (cut after 1.6 s) of the first six PCs. B: bottom score position; T: top score position; dotted line: 60% load; dashed line: 85% load, and full line: 95% load. ** significance at *p* < 0.01 between 60 and 85% as well as 60 and 95% loads; #, ## significance at *p* < 0.05 and *p* < 0.01 between 85 and 95% loads.

The first 8 PCs explained 98% of the variance between posture vectors. Reliability of scores over time (ICC) revealed good to excellent for the first four components (0.99–0.89) and decreased with higher components due to individual outliers (average measure in PC8:0.45 for 60% lifts, 0.78 for 85% lifts and 0.61 for 95% lifts) (Supplementary Table 1 and Supplementary Videos 3, 4) describe and illustrate what aspect of posture-change dominated the first 8 PC-movement components. The time-evolution of the scores is shown in Figure 3 for the first 6 PCs and significant differences (non-overlapping Cousineau-Morey confidence intervals) between load conditions were found in the first eight PC components. The range of motion (evaluated through minima and maxima) on the PC-axes did not differ between barbell loads, with two exceptions: squat position deepened [minima in PC 2, *F*(2, 7) = 6.18, *p* = 0.028, η*_*p*_*^2^ = 0.638] and elbow position was lower for higher loads [seen in PC6 scores at the deepest barbell position, *F*(2, 7) = 7.03, *p* = 0.021, η*_*p*_*^2^ = 0.668, Figure 3].

**FIGURE 4:**
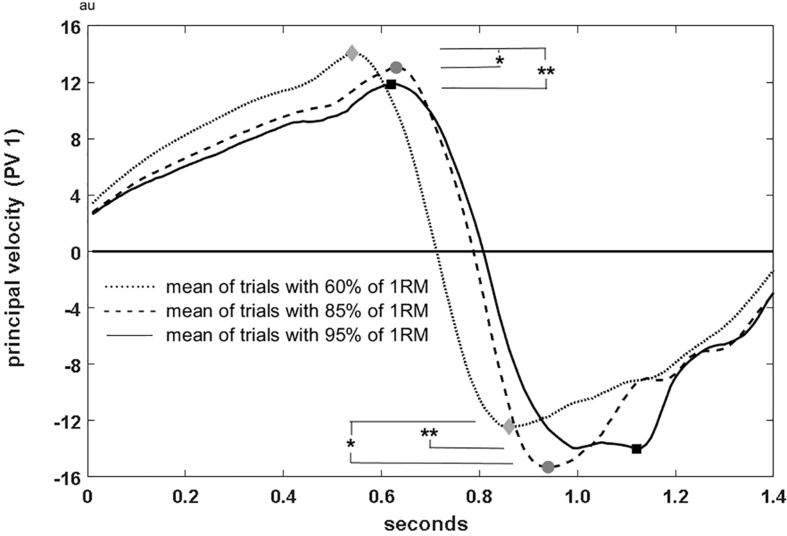
Postural velocity of PC 1 for 60, 85, and 95% of 1 RM load. *, ** significance at *p* < 0.05 and *p* < 0.01.

Maxima of postural velocities for the first component (PV1) in upward direction differed between the load conditions [*F*(2, 7) = 12.08, *p* = 0.005, η*_*p*_*^2^ = 0.775] as well as in downward direction [*F*(2, 7) = 9.04, *p* = 0.011, η*_*p*_*^2^ = 0.721] (Figure 4). Different speed characteristics (without figure) were also observed in PV2 showing higher speeds for higher loads in the turnover phase [*F*(2, 7) = 8.61, *p* = 0.013, η*_*p*_*^2^ = 0.711]. Lower PV3-speed revealed slower trunk raising [PV3 maxima *F*(2, 7) = 7.45, *p* = 0.018, η*_*p*_*^2^ = 0.680], and lower PV6 speed revealed slower downward elbow motion [PV6 minima: χ^2^(2) = 6.89, *p* = 0.032] for heavier barbell loads.

Postural acceleration components differed for PA3 [trunk raising; *F*(2, 7) = 9.13, *p* = 0.011, η_*p*_^2^ = 0.723] with highest acceleration found for lighter loads, and for PA6 (head, elbow, knee positioning) in 2nd pull phase and turnover, with highest acceleration found for heavy loads [*F*(2, 7) = 5.95, *p* = 0.031, η*_*p*_*^2^ = 0.629].

## Discussion

As a first result, the current study provided clear evidence for barbell relative load affecting the motion pattern of the clean in weightlifting: both data analysis approaches resulted in statistically significant load effects, on the variations between whole trials (PCA^trial^ approach), as well as on the individual movement components (PCA^posture^ approach). Secondly, the comparison of data analysis approaches highlighted their advantages and limitations. In brief, the PCA^trial^ approach required an additional pre-processing step (time-normalization), but was otherwise easy to implement. It proved sensitive for specific load effects, but covered a lower fraction of the overall variance in the data and did not reveal timing and speed differences that were detectable with the alternative method. An important advantage was that the observed differences in movement patterns could more easily be visualized and interpreted. On the other hand, the PCA^posture^ approach (based on synchronously executed movement components, PMs) proved to be more sensitive for a larger number of specific effects. However, due to the PM-based approach, it is more difficult to determine how the movement as a whole is affected, therefore requiring a deeper understanding of the movement technique when interpreting the results.

Specifically, PCA^trial^ revealed (Supplementary Videos 1, 2 and Figure 2) that with increasing relative barbell weights athletes (1) rose to a higher body elevation in the pull before dropping into the squat; (2) jumped into a wider stance during the squat; (3) went into a deeper squat; (4) kept their elbow lower during the squat and the subsequent rising. Parts of these findings agree with earlier studies investigating barbell position (which in Figure 2 can be derived from wrist marker positions). Ammar et al. (2018) reported decreasing vertical bar displacement and decreasing minimal bar height for loads between 85 and 100% of 1 RM. Hadi et al. (2012) reported the highest barbell displacement with 60% compared to 80 and 100% of 1 RM. Our study revealed that the upper body elevation was higher for higher loads—presumably a strategy to longer apply force onto the barbell in upward direction, thus creating a safer bar height before initiating the turnover phase. And also in agreement with earlier studies, we found lower squat positions for heavier loads. During squatting, our athletes jumped into a wider stance position for heavy loads—a position providing better stability and also allowing for a lower hip position as the athletes resume bearing the barbell load after the turnover. A barbell load effect on hip and knee extension (PC5 in our study) has also been reported in the literature (Kim et al., 2019; Kipp, 2020), while the accompanying difference in elbow positioning at the end of the catching phase (Figure 2C) has not been documented before. Interestingly the highest elbow position was seen in the 60% lifts where the risk of “losing” the bar would be lowest. A reason might be that force application is easier in a lower elbow position, or that it is an unconscious safety strategy that allows athletes to faster free themselves of the barbell load in case of difficulties during the catching phase. Our results do not agree with findings of Kipp et al., 2012, who reported no effect of external load (65, 75, and 85% of 1 RM) on kinematic movement synergies considering hip, knee, and ankle joints.

PCA^posture^ revealed significant differences in score waveforms over time in all 8 PCs between different loads (Figure 3 shows the first six). Extracted features of the scores and of their derivatives showed significant results for PC1, PC2, PC3, and PC6 representing the body elevation, body positioning under the bar, trunk raising and elbow positioning respectively. The significant differences in the minimum scores of PC2 (lowest squat position for highest loads) and in the scores of PC6 (e.g., elbow positioning in the turnover phase) at lowest barbell position correspond well with the findings of PCA^trial^. Further, PV calculation revealed higher maxima in PV1 (body elevation movement speed) for lower loads and highest PV1 in the catching phase for 85% lifts. This might demonstrate that catching the bar in 95% lifts provokes earlier resistance to the falling bar und seems to be more effective for squat position control, which does not seem to play a role for lighter loads. Significant differences in the maximum of PV3 (trunk raising, pelvis tilting) suggest a different timing structure in the pull phases between 60, 85, and 95% lifts. A similar observation was mentioned by Enoka (1988), who indicated adaptation of the temporal sequence with increasing load. Results in PV3, also correspond to the findings of Sandau and Granacher (2020), who reported the highest loss in vertical bar velocity during a snatch was seen in the first pull when increasing loads from 70 to 100% of 1 RM. Finally, considering acceleration patterns, we can compare our results with Kipp and Harris (2015), who analyzed vertical barbell acceleration patterns and reported that a more steady acceleration pattern of the barbell in the second knee bend and second pull phase leads to higher relative loads in maximal snatch lifts. Thus, acceleration patterns leading to this desired technical feature are of special interest. In our study, 60% lifts differed significantly from the 85 and 95% lifts in peak acceleration in two of the eight movement components. This might indicate that lifting lighter loads does in fact cause a deviation from the desired adaptations for load maximization.

### Limitations

Limitations of the current study include a small sample size and that no *a priori* power analysis was conducted—in this sense, the current study could be seen as a pilot study. Nevertheless and despite conservative corrections for error accumulations (Bonferroni), we still observed significant effects of the relative load. We are therefore confident that our data provides reliable support for the main hypothesis.

Second, the study included a diverse population of weightlifting athletes, from junior level to internationally competing adult athletes. Our results may therefore not be generalizable to elite athletes and may not reflect elite lifting technique. Then again, our findings are for the same reason better applicable to the training of young athletes, where the investigated research question is very relevant.

Third, wrist movements are a potentially interesting aspect of weightlifting technique, however, the analyzed marker set was not well suited to reveal wrist movements: the marker on the finger frequently fell off and could therefore not be included in the analysis, whereas the barbell markers, due to bending of the bar caused by loads up to 110 kg, also did not provide direct information on hand positioning. Wrist movements in the context of weightlifting technique should therefore be investigated in future studies.

## Conclusion and Implications for Practitioners

The current study supported the hypothesis that kinematic patterns of the clean are affected by barbell weight. For the coach or athlete, the current study provides a list of features that are affected by heavier loads, specifically, higher body elevation relative to the bar before and deeper position in squatting, accompanied by wider standing position and less elevated arms; lower velocities of associated movement components; and changed postural acceleration characteristics especially in the turnover phase. This suggests e.g., that moving under the barbell as quickly as possible has high relevance in technique training and is worth to be addressed separately.

The stick-figure animations created in the current study combine information from all volunteers and the PC-scores allow for statistical testing. For an athlete or coach they provide an objective tool for technique assessments, unlike, for example, classical video analysis where only the individual technique of one athlete at a time can be assessed, or where individual athletes are compared to each other, but cannot be compared to whole groups.

For researchers, the current study suggests that PCA applied to a matrix of time-normed marker trajectories (PCA^trial^) better reveals local adaptations within the movement execution. PCA applied to a matrix of posture positions (PCA^posture^) reveals coordination patterns of the movement and facilitates an analysis of timing, speed and acceleration differences in the movement execution.

## Data Availability Statement

Data are published on figshare https://doi.org/10.6084/m9.figshare.13333937.v1.

## Ethics Statement

The studies involving human participants were reviewed and approved by the Board for Ethical Questions in Science of the University of Innsbruck (Certificate 42/2015). The patients/participants provided their written informed consent to participate in this study. Written informed consent was obtained from the individual(s) for the publication of any potentially identifiable images or data included in this article.

## Author Contributions

NS and PF conceived the study. NS conducted all experiments. IW, NS, and PF analyzed the data. FW provided the visualizations. IW and NS created the first draft for the manuscript. IW, NS, FW, and PF revised and finalized the manuscript. All authors contributed to the article and approved the submitted version.

## Conflict of Interest

NS was employed by company BFF Training Ulm. The remaining authors declare that the research was conducted in the absence of any commercial or financial relationships that could be construed as a potential conflict of interest.

## Footnotes

1 https://www.uibk.ac.at/isw/forschung/neurophysiologie-bewegungswissenschaft/software.html

## Supplementary Material

Supplementary Table 1 Description, explained variance and relative barbell load effects for the first 8 PC components in both analysis approaches.

Supplementary Video 1 Animated stick figure representation of the effects of relative barbell load on the execution of PC1 in the PCA^trial^.

Supplementary Video 2 Animated stick figure representation of the effects of relative barbell load on the execution of PC5 in the PCA^trial^.

Supplementary Video 3 Animated stick figure representation of PM1–PM4 resulting from PCA^posture^.

Supplementary Video 4 Animated stick figure representation of PM5–PM8 resulting from PCA^posture^.
