# Supplementary material for: How Do Movement Patterns in Weightlifting (Clean) Change When Using Lighter or Heavier Barbell Loads?—A Comparison of Two Principal Component Analysis-Based Approaches to Studying Technique
Source: Front Psychol. 2021 Jan 25;11:606070. doi: 10.3389/fpsyg.2020.606070 (PMC7868553; doi:10.3389/fpsyg.2020.606070)
Supplement: Supplementary Table 1 — Description, explained variance and relative barbell load effects for the first 8 PC components in both analysis approaches. [file Table_1.DOCX]

Supplementary Material

# Supplementary Table

Table 1: description, explained variance and relative barbell load effects reaching significance for both PCA approaches

| ***PC k*** | ***Description of component k*** | ***explained variance*** | ***effect of relative barbell load*** |
| --- | --- | --- | --- |
| ***PCA trial*** |  |  |  |
| **PC 1** | waveform difference in body elevation and squat position | 33.4 % | score difference |
| **PC 2** | difference in upper body elevation and leaning backward | 20.6 % |  |
| **PC 3** | difference in squat, jumping phase, foot position | 8.5 % |  |
| **PC 4** | difference in head position and squat depth | 7.6 % |  |
| **PC 5** | difference in knee stretch during the second pull phase and elbow positioning in catching phase | 4.9 % | score difference |
| **PC 6** | difference in leaning backward in second pull phase | 4.1 % |  |
| **PC 7** | difference in jumping phase and foot position | 3.1 % |  |
| **PC 8** | difference in the turnover phase | 2.6 % |  |
| ***PCA posture*** |  |  | max / min of |
| **PC 1** | body movement upward, broadening stand position | 65.0% | velocity |
| **PC 2** | body movement relatively to the bar, feet apart | 29.0% | scores, velocity |
| **PC 3** | trunk raising, pelvis position, rotation of body in relation to the bar | 1.7 % | velocity, acceleration |
| **PC 4** | positioning of elbows, bar position towards the body | 1.4 % |  |
| **PC 5** | back and forward positioning of the body to the bar | 0.7 % |  |
| **PC 6** | second pull and turnover with head, elbow, knee, heel positions | 0.4 % | velocity, acceleration |
| **PC 7** | elbow upward movement in pulls, foot axis rotation | 0.3 % |  |
| **PC 8** | hip movement and knee bend during pulls | 0.2 % |  |
